# Empowering CKD and Hemodialysis Patients with mHealth: Implementation of the NephroGo App in Europe

**DOI:** 10.3390/jcm14176219

**Published:** 2025-09-03

**Authors:** Giedrė Žulpaitė, Karolis Vyčius, Urtė Deinoravičiūtė, Edita Saukaitytė-Butvilė, Laurynas Rimševičius, Marius Miglinas

**Affiliations:** 1 Clinic of Gastroenterology, Nephrourology and Surgery, Institute of Clinical Medicine, Faculty of Medicine, Vilnius University, M. K. Ciurlionio Str. 21, LT-03101 Vilnius, Lithuania; 2 Independent Researcher, LT-02196 Vilnius, Lithuania; 3 Institute of Applied Mathematics, Vilnius University, Naugarduko Str. 24, LT-03225 Vilnius, Lithuania; 4 Public Institution Antakalnis Outpatient Clinic, Antakalnio Str. 59, LT-10207 Vilnius, Lithuania

**Keywords:** chronic kidney disease, mobile application, patient self-management

## Abstract

**Background/Objectives:** Chronic kidney disease (CKD) requires intensive dietary and lifestyle management, yet patient engagement and access to tailored education remain limited, particularly outside clinical settings. This study describes the development and implementation of NephroGo, and evaluates its usability, user engagement, and perceived acceptability among patients with CKD. **Methods**: The app was developed based on clinical and dietary guidelines, incorporating personalized nutrient recommendations, dialysis tracking, and educational content. Technically, it features a Django backend, Flutter mobile frontend, and secure cloud-based hosting. User feedback was collected through one-time interviews (*n* = 10) and a standardized Mobile App Rating Scale (MARS) survey (*n* = 32). Longitudinal usage data over four years were also analyzed. **Results:** Initially, NephroGo was downloaded by 204 users, of whom 93.6% were considered active users based on defined behavioral engagement thresholds. Over a four-year period, the app accumulated a total of 1670 downloads. This study focuses on evaluating user engagement, usability, and perceived acceptability of the NephroGo app over a four-year period. Most users were female (52.3%) and aged 30–65. Stage 5 CKD patients and those undergoing peritoneal dialysis (PD) had the highest engagement. The most-used feature was the personalized nutrition calculator, with sodium being the most frequently exceeded nutrient. The average MARS score was 4.09 ± 0.66, with functionality rated highest (4.27 ± 0.74). App ratings were significantly higher among users referred by physicians (*p* = 0.039). **Conclusions**: NephroGo offers a scalable digital tool to support dietary management and health monitoring, with potential to complement standard nephrology care in a resource-conscious manner.

## 1. Introduction

Chronic kidney disease (CKD) is a rapidly growing global public health concern. According to the World Health Organization, approximately one in ten adults worldwide is affected by kidney disease [1]. The Global Burden of Disease Study estimates that nearly 10 million people die annually due to kidney-related conditions, marking a 32% increase over the past decade [2]. The number of individuals suffering from CKD rises by an estimated 8% each year, thereby intensifying its social and economic burden across healthcare systems globally [3]. Given this trajectory, the prevention and management of CKD must be prioritized within the healthcare strategies of developed nations. Early diagnosis and patient engagement are essential elements in mitigating disease progression. However, a CKD diagnosis often demands significant lifestyle adjustments, including adherence to complex medical regimens and strict dietary restrictions. The American Society of Nephrology emphasizes that dietary control remains one of the most challenging aspects for CKD patients [4,5]. Compounding this challenge is the general lack of transparency in food labeling, particularly regarding detailed nutrient content such as sodium, potassium, and phosphorus levels.

Numerous studies have underscored a widespread deficiency in patient knowledge related to CKD, significantly contributing to poor compliance with treatment recommendations [5]. Although structured educational sessions are typically provided in clinical settings, these are often insufficient for long-term disease management. Periodic retraining has improved outcomes, but the availability of healthcare personnel, time, and financial resources are limited [6]. This issue became especially critical during the COVID-19 pandemic when access to in-person training was further restricted.

In this context, mobile health (mHealth) technologies offer a scalable, cost-effective solution to improve patient education and engagement [7]. NephroGo, a multifunctional mobile application developed for CKD patients, addresses this unmet need. It is currently the only app in Europe explicitly designed to assist patients in managing their condition through personalized nutrient tracking, self-management education, health and PD monitoring. NephroGo offers a practical and affordable tool to support patient empowerment in CKD treatment, potentially alleviating the resource constraints faced by traditional healthcare systems.

Improved patient activation and self-management have been shown to reduce unnecessary healthcare utilization and associated costs by supporting better adherence to lifestyle changes such as diet and symptom monitoring—key aspects of CKD care [8]. In contrast, lower activation is linked to increased hospitalizations, poorer treatment adherence, and higher overall healthcare expenditures. These associations underscore the potential of mHealth tools to not only improve clinical outcomes but also alleviate the economic burden of CKD on healthcare systems [8,9].

This article presents the rationale, development, and early evaluation of the NephroGo mobile application. We discuss its potential to enhance patient self-management, improve treatment compliance, and reduce the burden on healthcare providers, thereby contributing to more effective and sustainable CKD care.

## 2. Materials and Methods

### 2.1. Application Development

The development of the NephroGo mobile application was guided by clinical and dietary recommendations from the National Kidney Foundation Kidney Disease Outcomes Quality Initiative (KDOQI) and the Academy of Nutrition and Dietetics. Based on these guidelines, we created algorithms to calculate personalized daily intake recommendations for potassium, sodium, phosphorus, protein, and water for patients with CKD.

To establish a comprehensive food composition database within the application, data were sourced from the Lithuanian Centre for Health Education and Disease Prevention and the Frida Food Data database (National Food Institute, Technical University of Denmark, Copenhagen, Denmark). The food composition database used in NephroGo is region-specific and tailored to reflect national dietary patterns; for example, the database differs between Lithuania, Germany, Austria, and Switzerland to ensure local relevance and accuracy. The app’s PD feature was developed using patient education materials from Vilnius University Hospital Santaros Klinikos, ensuring clinical relevance and usability for PD patients.

Patient needs and preferences were gathered through structured interviews and consultations with individuals living with CKD during the initial design phase, and their feedback informed the interface layout, content, and overall functionality of the application.

### 2.2. Technical Implementation

We developed the backend of the NephroGo application using Django, a Python 3.12-based web framework. A relational data model was implemented with PostgreSQL, ensuring secure and structured data storage. The application programming interface (API) was designed following the REST architectural style, using JSON for data exchange and the Google Brotli algorithm for compression during transmission. The API is fully compatible with the OpenAPI specification, ensuring standardization and ease of integration.

Django Admin was employed for application administration, while JSON Web Tokens (JWT) were used to manage secure user authentication and access control.

We developed the mobile front-end of NephroGo using Flutter, allowing for cross-platform deployment on both Android and iOS. Both mobile applications communicate with the central server through the NephroGo API and adhere to Material Design principles to maintain a consistent and user-friendly interface. User registration and login via Google and Facebook were implemented using Firebase Authentication. Deployment to Google Play and Apple App Store was fully automated using GitHub Actions, facilitating efficient version control and continuous integration.

In addition to the mobile applications, we created a static website using the Hugo static site generator, supporting both Lithuanian, English and German versions available at www.nephrogo.lt and www.nephrogo.com, respectively. The websites are hosted via Firebase Hosting, and continuous integration (CI) workflows were implemented using GitHub Actions to streamline updates and deployment.

The application is currently available in Lithuanian, German, and English, ensuring accessibility for users in the regions where the app is deployed.

Key steps in the development of NephroGo include the integration of clinical guidelines, algorithm-based personalization, database compilation, secure technical implementation, and cross-platform deployment.

### 2.3. User Testing and Evaluation

Two months after launch, we administered an online cross-sectional survey to a broader group of users (*n* = 32). The survey included demographic questions and incorporated the validated Mobile Application Rating Scale (MARS), which assesses app quality across six domains: engagement, functionality, esthetics, information quality, subjective quality, and app-specific items. Each domain was rated using a 5-point Likert scale, with higher scores indicating better-perceived performance.

This mixed-methods approach allowed for qualitative insights and standardized quantitative evaluation of the app’s usability, appeal, and user experience.

### 2.4. Data Analysis

Data from the survey were processed and analyzed using Microsoft Excel and R: a language and environment for statistical computing. Quantitative results from the MARS evaluation were used to assess user satisfaction and app performance in a standardized and reproducible manner. Descriptive statistics of app users’ data was calculated using R Core Team (2021) [10]. A significance level of *p* < 0.05 was used to determine statistical significance in all analyses.

### 2.5. Ethical Considerations

This study did not involve any intervention or identifiable personal data and was therefore exempt from ethical committee approval.

### 2.6. Use of Generative AI

No generative artificial intelligence (GenAI) tools were used to generate, analyze, or interpret data. This manuscript was written and edited without AI’s assistance, creating substantive content.

## 3. Results

**Figure 1:**
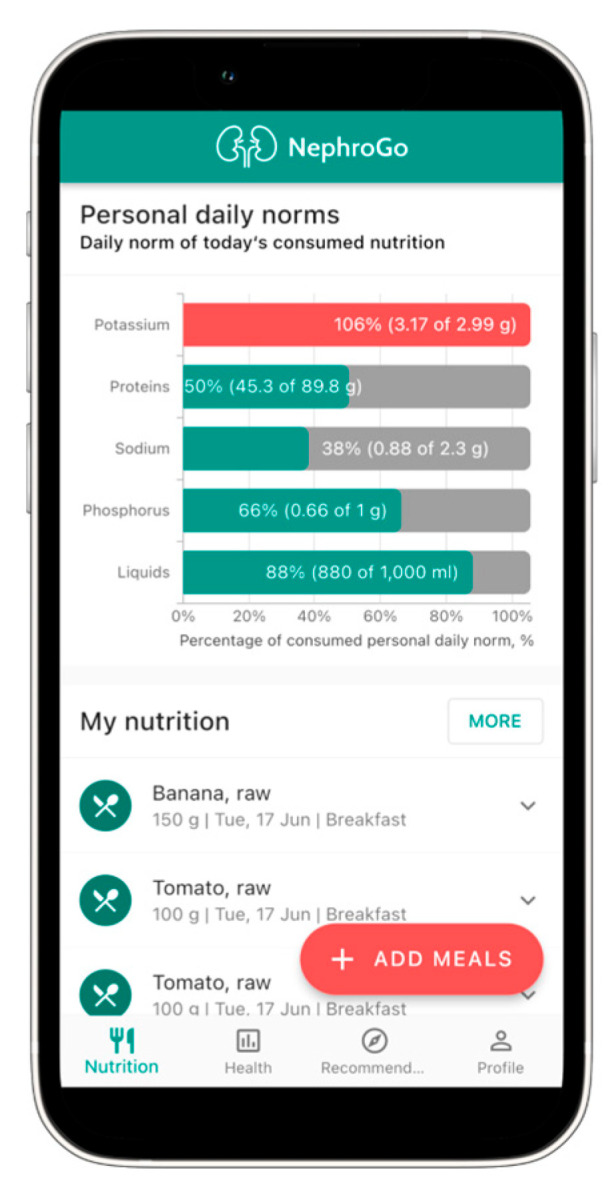
The user interface of the NephroGo mobile application: nutrition calculator.

**Figure 2:**
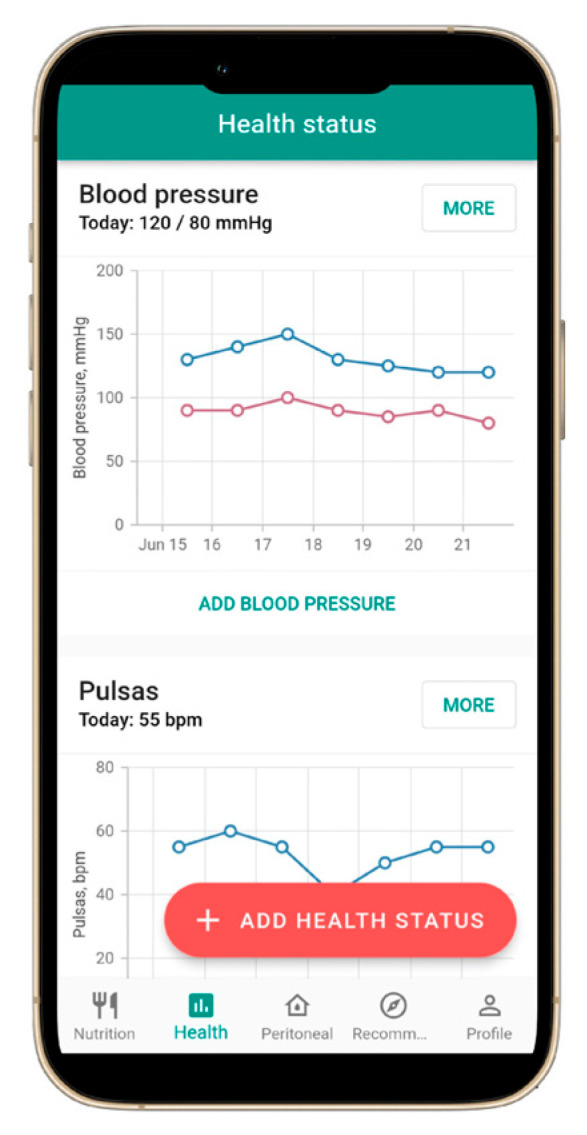
User interface of the NephroGo mobile application: health status tracking.

**Figure 3:**
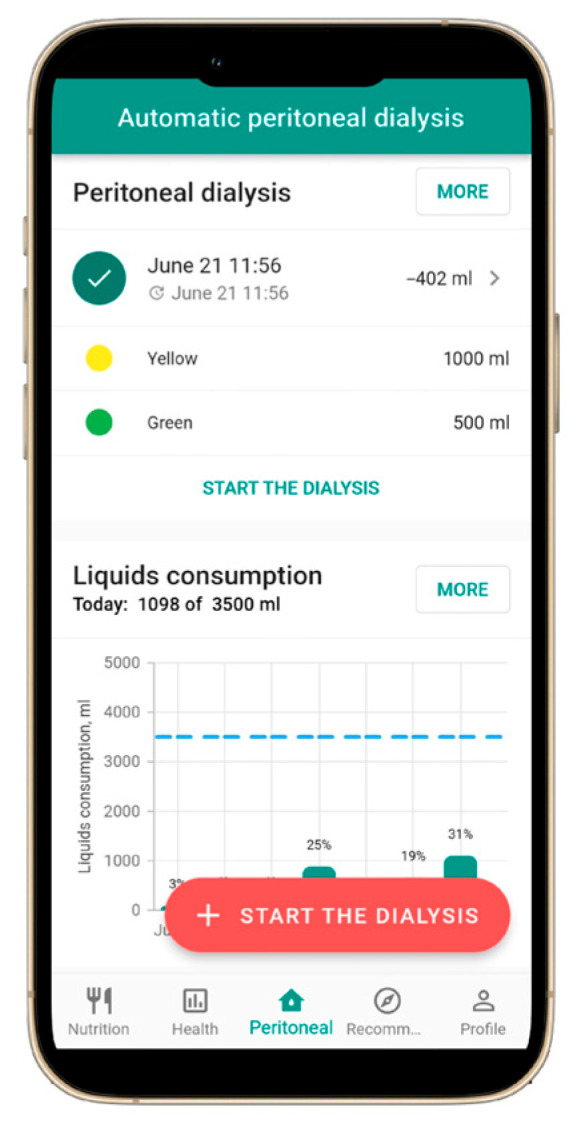
The user interface of the NephroGo mobile application: PD function.

**Figure 4:**
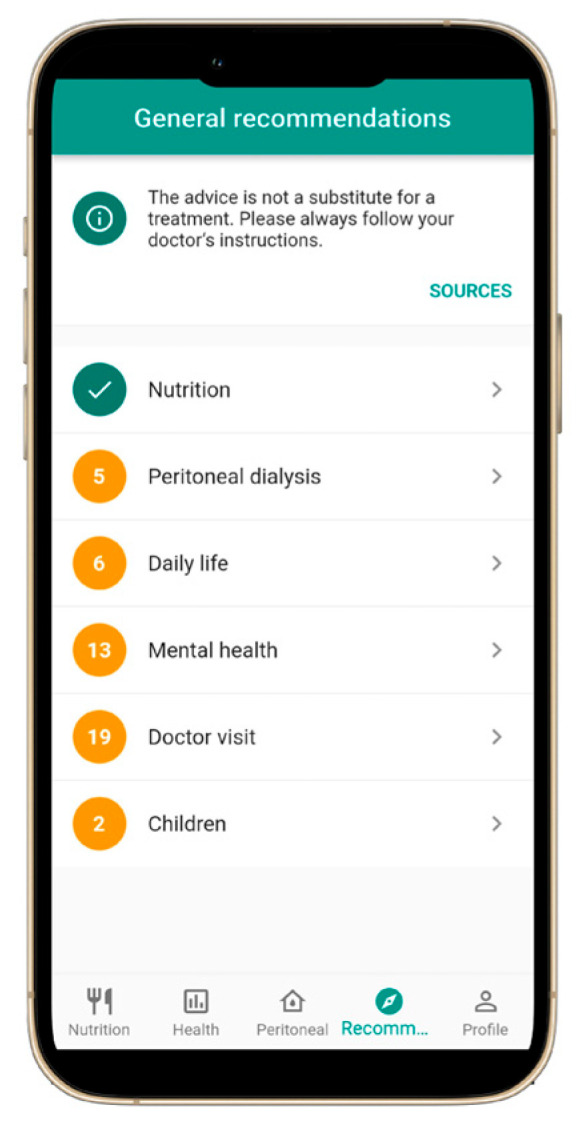
The user interface of the NephroGo mobile application: general lifestyle and medical recommendations function.

A mobile application specifically designed for patients with CKD, including those undergoing dialysis, was successfully developed and named NephroGo. The application comprises four primary functional modules: a personalized nutrition calculator (Figure 1), health indicator tracking (Figure 2), PD management (Figure 3), and an educational advice section (Figure 4).

### 3.1. Nutrition Calculator

The personalized nutrition calculator allows users to search for food items within the unique NephroGo database and view detailed nutritional information, including levels of potassium, sodium, phosphorus, protein, carbohydrates, fats, fluids, and kilocalories. Based on individual user data and clinical dietary guidelines, the application automatically calculates the recommended daily intake of these nutrients. The results are presented in a user-friendly graphical and numerical format, enabling patients to understand and track their dietary adherence easily. The application also generates daily, weekly, and monthly summaries, helping users monitor their compliance with kidney-friendly dietary recommendations (Figure 1). Recommendations for mineral intake are adjusted based on the stage of CKD, allowing more flexibility in earlier stages where kidney function is better preserved.

### 3.2. Health Indicator Tracking

NephroGo includes a feature that allows users to record and monitor various daily health parameters in one convenient location. These indicators include blood pressure, pulse, body weight, urine output, blood glucose concentration, and the severity of symptoms such as edema and shortness of breath. The app visualizes these indicators’ trends using graphs and numerical values, enabling users to recognize significant changes early. Patients are encouraged to share these logs with their healthcare providers during consultations, improving communication regarding daily health status and treatment efficacy. At present, the application does not provide real-time feedback or alerts; instead, users can access educational content within the app that informs them about target blood pressure ranges and the implications of elevated readings. Future updates of the application are planned to incorporate automated alerts that notify users when tracked health indicators, such as blood pressure, exceed recommended thresholds.

### 3.3. Peritoneal Dialysis Support

Although NephroGo is suitable for all CKD patients, it also includes a specialized module for patients PD. The application supports automated peritoneal dialysis (APD) and continuous ambulatory peritoneal dialysis (CAPD). Users can input all dialysis-related data, and the app automatically calculates fluid balance. It also generates detailed dialysis summaries, which can be shared with healthcare professionals to support clinical decision-making.

### 3.4. Educational and Support Section

The fourth core feature of NephroGo is a dedicated advice section, offering educational content on topics such as nutrition, dialysis procedures, and various aspects of daily life with CKD. This function includes guidance on physical activity, travel, sexual health, work, and study. Additionally, the section provides emotional support resources to address the psychological challenges associated with living with a chronic illness (Figure 4).

Together, these features position NephroGo as a comprehensive and patient-centered digital tool for the self-management of CKD.

### 3.5. User Characteristics and Early App Performance Feedback

Initially, 204 patients with CKD downloaded the NephroGo mobile application. Of those, 93.6% (*n* = 191) actively used the application. Active users were defined as those who used the app’s key features—such as the food tracking function—at least twice. Within this group, engagement levels were notably high: users logged an average of seven food items per day and consistently entered at least one health indicator daily for a full week. The majority of users were female (*n* = 138, 67.6%). More than half of the users had been living with CKD for over 10 years (*n* = 111, 54.4%), and a quarter of them were in stage 5 CKD (*n* = 52, 25.5%) (Table 1).

A significantly higher proportion of users were not receiving any form of renal replacement therapy (*n* = 81, 39.7%), while one-third were undergoing hemodialysis (*n* = 59, 28.9%), and only a small number were treated with PD (*n* = 13, 6.4%) (Table 1).

The most frequently used feature of the NephroGo app was the personalized nutrition calculator, with 66.2% (*n* = 135) of users engaging with it daily. On average, users added seven food items per day (±1). The most common dietary deviation was excessive sodium intake (58, 30.2% of users), while exceeding daily caloric intake was the least common (12, 6.3% of users) (Table 1).

A total of 32 users participated in a one-time quality evaluation survey of the NephroGo application. Nearly half reported learning about the app from healthcare professionals (*n* = 14, 43.8%), while over one-third discovered it through social media (*n* = 11, 34.4%). Most respondents were urban residents (*n* = 26, 81.3%), and more than half had higher education degrees (*n* = 18, 56.3%) (Table 2(1)).

The overall quality rating of “NephroGo” on the MARS (Mobile App Rating Scale) was high, with an average score of 4.09 ± 0.66. The highest-rated dimension was functionality (4.27 ± 0.74), while engagement received the lowest rating (3.81 ± 0.80). App ratings did not significantly differ based on users’ sex, education level, or place of residence (Table 2(2)).

Statistically higher ratings were observed among users undergoing PD (5.00 ± 0.00, *p* = 0.0089) and patients with stage 5 CKD (4.52 ± 0.52, *p* = 0.0016). Additionally, users whom a physician had recommended the app rated it significantly higher (4.50 ± 0.68, *p* = 0.039) (Table 2(2)).

### 3.6. Long-Term User Characteristics and Feature Engagement

After 4 years, we analyzed longitudinal app usage data of the users. Slightly more females (52.3%) than males (47.7%) have downloaded and used the app, which implies greater health-tracking engagement among women (Table 3). The table shows a notable concentration of users around age 30 (22.9%), which is likely due to the app’s default birthdate setting of 1 January 1995. This results in a data conglomeration across four years, reflecting default input rather than users’ actual age. Excluding this outlier group, the data indicate higher usage among middle-aged and older adults. This aligns with the chronic nature of CKD and supports the observation that the app is primarily used by individuals within the typical age range affected by the disease (Table 3). Following the development of the app for adult users, we received requests from a community of parents of children with CKD to create a pediatric NephroGo version. In response, a modified version was launched, resulting in 36 users aged 0–18 years, where the app was used with parental consent and supervision (Table 3). Most users are either in Stage 5 (27.2%) or have not reported their CKD stage (26.3%). This data suggests the app is attracting individuals at more severe stages of kidney disease or those lacking regular medical follow-up. Intermediate stages (Stage 3 and Stage 4) also show a significant portion of users, reflecting broad clinical engagement. The most significant proportion of users have been living with kidney disease for over 10 years (31.1%), closely followed by those with less than 1 year (27.8%) and 1–5 years (27.4%). This data suggests the app appeals to newly diagnosed patients and those with long-term management needs, possibly reflecting the tool’s relevance across the disease trajectory (Table 3). Most users (57.0%) have not undergone dialysis, suggesting they are either pre-dialysis or post-diagnosis but conservatively managed. Among those who do receive dialysis, hemodialysis is most common (23.8%), followed by manual PD (8.9%) and automatic PD (4.0%). A notable 6.2% are post-transplant patients. The majority of users (82.8%) do not have diabetes. Among those who do, Type 2 diabetes is more common (9.5%) than Type 1 diabetes (7.4%). Only a small fraction of entries (0.3%) have unknown diabetes status, indicating relatively complete data in this field (Table 3).

**Figure 5:**
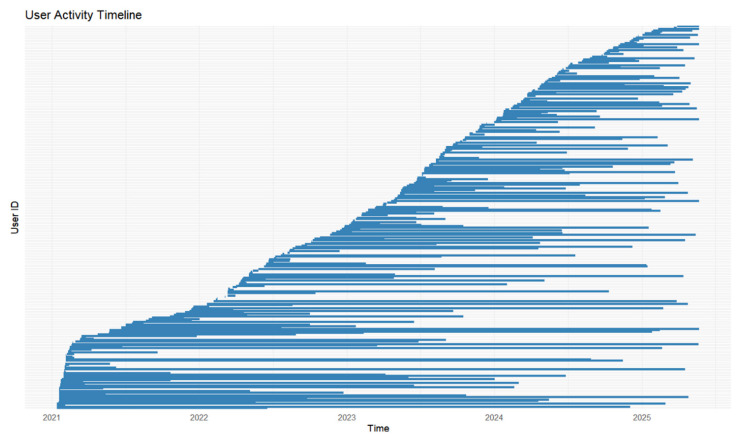
Duration of use for active users.

**Figure 6:**
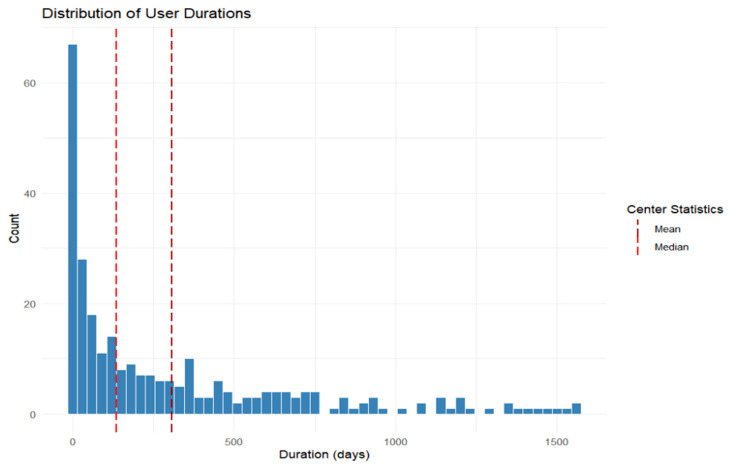
Use durations’ distribution.

From this point on, we analyze only the behavior of app’s most active users. 4% of people who downloaded the app used the food tracking feature actively, 2% used peritoneal dialysis tracking and 13% used daily health tracking. Figure 5 indicates the duration of use for all active users of the app. The duration of use was determined by taking the difference between the last log in and the first one. We can see that more users who actively use the app join in the first half of a year, but the duration of use is longer on average for those who join later in the year. Furthermore, the distribution of use times (Figure 6) indicate the mean usage of 302 days and median of 136 days for active users. The usage times do not follow normal distribution, has a long right tail, this together with small volume of data creates difficulties for in depth analysis.

Food tracking activity was more common among female users, who accounted for 59.1% of entries, compared to 40.9% by male users. Although males make up nearly half of the overall user base, this suggests that female users were more likely to engage with the dietary tracking feature (Table 4). In terms of CKD stage, the largest proportion of food-tracking entries came from users in Stage 5 (39.4%), followed by those with an unknown stage (30.3%). This pattern indicates that patients with more advanced kidney disease, or those without clear staging, may be particularly motivated to monitor their dietary intake—possibly due to greater clinical need or increased awareness of dietary risks (Table 4).

Looking at the peritoneal dialysis tracking feature, out of 614 users who registered that they use some kind of dialysis, 28 (4%) actually entered dialysis data at least twice. Male users account for 67.9% of PD-tracking entries despite making up a smaller proportion of overall users. This data suggests that male users who engage with the app are more active or consistent in logging their dialysis treatments, potentially reflecting different self-management behaviors (Table 5). Most dialysis tracking comes from users aged 41–60 (46.5%), followed by entries with younger age 21–40 (25%). Middle-aged users are the most consistent in documenting their dialysis routines, possibly due to greater digital literacy or self-care involvement.

Female users accounted for a higher proportion of daily health tracking entries (55.1%) compared to male users (44.9%), indicating greater engagement in regular health self-monitoring among women (Table 6). The most active age group in health tracking was 41–60 years (35.6%), followed by users aged 21–40 (25.5%) and 61–80 (21.3%), suggesting that middle-aged adults are the most consistent in monitoring daily health indicators. In terms of CKD stage, users in Stage 5 contributed the largest proportion of entries (45.5%). This pattern suggests that individuals with more advanced kidney disease may be more engaged in daily health tracking, likely due to increased clinical monitoring needs and a greater awareness of their condition’s progression.

Sodium is the most commonly exceeded nutrient, followed by liquids. This information highlights sodium and fluid management as potential focus areas for user education or app alerts.

## 4. Discussion

This study provides the first large-scale evaluation of a mobile health (mHealth) application developed in Europe specifically for individuals with CKD. The app demonstrated high user engagement (with 93.6% of those who downloaded the app actively using it) and strong satisfaction scores on the Mobile App Rating Scale (MARS) (mean score 4.09 ± 0.66). These findings suggest that NephroGo is a practical and acceptable tool for supporting self-management in many CKD patients.

### 4.1. Clinical Utility and Feature Engagement

Managing diet in patients with CKD is a complex and often overwhelming task [11]. Patients must frequently adapt to changing dietary requirements, while healthcare providers face ongoing challenges in delivering sustained and personalized nutritional education [4,5]. These barriers underscore the need for scalable, supportive tools that enhance self-management and complement the work of dietitians and nephrology teams [11,12].

Among NephroGo’s features, the personalized nutrition calculator was frequently used, with users logging multiple food items daily. This consistent engagement suggests a strong user demand for tools that simplify complex dietary planning [11]. Notably, sodium was the most commonly exceeded nutrient, echoing previous findings that sodium restriction remains one of the most challenging aspects of dietary adherence in CKD care [13]. This recurring pattern highlights an important and persistent challenge in patient self-management that merits further clinical attention.

Unlike general diet-tracking apps, NephroGo provides stage-specific nutrient targets, helping to reduce confusion often experienced by CKD patients navigating shifting dietary advice. This tailored guidance is particularly valuable in conditions like CKD, where recommendations vary depending on disease progression [14]. The app’s individualized dietary feedback reinforces evidence that tailored nutrition support improves adherence and self-efficacy. These findings support the role of condition-specific digital tools in bridging gaps between clinical visits and in sustaining long-term dietary behavior change [15].

### 4.2. User Characteristics and Behavior Patterns

#### 4.2.1. CKD Stage

The results also shed light on user demographics and behaviors. Our results showed strong engagement among Stage 5 CKD patients. This suggests that some individuals at advanced disease stages are motivated and capable of managing their condition using digital tools—especially those already on dialysis or in regular contact with healthcare providers. However, this contrasts with findings from a cross-sectional study, which reports low patient activation in Stage 5 CKD, especially among older adults and those not yet on dialysis [8,16]. The discrepancy may reflect differences in population characteristics: our data likely capture users who are already more digitally literate, health-aware, or further along in their treatment planning, whereas the cross-sectional study focused on a broader and potentially more vulnerable pre-dialysis cohort [8,17]. The comparison underscores an important challenge in mHealth. While digital tools may effectively support those already engaged, they may not reach individuals with lower activation levels at risk of poor outcomes and higher healthcare utilization. This highlights the need for stratified or tailored approaches in app design and clinical implementation to support varying readiness levels for self-management. In this context, digital health tools should not be viewed as one-size-fits-all solutions but as part of a broader, patient-centered strategy to improve activation across the CKD continuum.

#### 4.2.2. Source of Recommendation

Notably, the source of app recommendation had an impact: users who were introduced to the app by their physicians rated it significantly higher, underscoring the influential role healthcare professionals play in digital health adoption. This finding aligns with prior research stating that patients are more likely to adopt and trust digital health tools when recommended by a healthcare provider [8]. When a doctor suggests using a digital health tool, it can significantly increase a patient’s willingness to adopt and engage with that technology [18].

#### 4.2.3. Sex

We also observed sex-related trends. Although our study did not aim to assess sex-based differences statistically, some usage patterns by sex were observed. Women represented a slight majority of total users, while men appeared more active in logging PD data. This mirrors previous research suggesting that male users, once engaged, maybe more consistent in disease-specific tracking, whereas women are more commonly engaged in general health app use [19]. Social and behavioral factors, including caregiving responsibilities and perceived health risks, may influence these differences [20]. Interestingly, dietary tracking was relatively balanced between sexes in our study, differing from prior findings that women tend to lead in nutritional logging. This may indicate that NephroGo’s user-centered design helped reduce sex-related barriers to dietary self-management. Moreover, previous research has shown that female sex is associated with higher levels of the knowledge, skills, and confidence needed to manage one’s healthcare—factors that are key to effective self-management. This may help explain the higher engagement observed among women in our user group [21].

#### 4.2.4. Age

In our study, app usage was highest among individuals aged 31–70, with the most represented age groups being 31–40 (16.6%), 51–60 (14.3%), and 61–70 (12%). This aligns with previous research showing that middle-aged adults are often the most active adopters of digital self-management tools due to higher digital confidence. Notably, we also observed a relatively good level of engagement among older users (71+). This may reflect the app’s intentionally simple design, prioritizing accessibility for users with lower digital literacy.

Previous studies have identified that older adults, particularly those over 65, often face challenges with digital health tools due to technical anxiety, limited experience, and frustration with complex interfaces [20,21]. Poorly adapted designs can further reduce motivation and usability in this group [22,23,24]. Our approach may have contributed to greater inclusivity and acceptance among older CKD patients by emphasizing intuitive navigation and minimizing technological barriers.

The application’s high usage and favorable evaluations suggest that NephroGo may serve as a scalable digital adjunct to traditional nephrology care, with potential to support efficiency and reduce resource burden. The feature-specific insights (e.g., nutrition calculator as most used; sodium most frequently exceeded) provide practical guidance for refining educational modules and alert systems within the app. Furthermore, the association between higher MARS scores and physician recommendation underscores the value of clinician engagement in digital health deployment, supporting the need to formally integrate such tools into care pathways.

#### 4.2.5. Strengths and Limitations

Compared to existing mHealth solutions for CKD, NephroGo is unique in its integration of stage-specific dietary tracking, PD support, and real-time visualizations. A key strength of this study is the inclusion of four years of real-world app usage data. Long-term engagement data are uncommon in mHealth research, particularly in nephrology. This extended time frame provides a more accurate picture of how patients use the app over time and supports its potential for long-term integration into chronic care.

However, several limitations must be acknowledged. First, the reliance on self-reported demographic data and app usage introduces potential biases. The uneven distribution of CKD stages, with a high proportion of “unknown” entries, suggests data entry quality or health literacy challenges. Secondly, outcome measures such as clinical biomarkers (e.g., creatinine, glomerular filtration rate (GFR), hospitalization rates) were not assessed, limiting insight into direct health impacts. This study did not assess longitudinal changes in nutritional intake, as users were not required to track their food items consistently over time, and input frequency varied widely across the user population. Lastly, the MARS survey sample (*n* = 32) was relatively small and included a higher proportion of PD users (40%) compared to the overall user base (6.4%), which may reflect greater engagement among this subgroup. As only a portion of the total users engaged deeply with all features, this may influence the extent to which findings reflect broader usage trends. While this provides valuable insight into highly active users, it may limit the generalizability of satisfaction scores to the broader population.

#### 4.2.6. Future Research Directions

Future studies should evaluate NephroGo’s clinical effectiveness on long-term health outcomes, including adherence, nutritional biomarkers, and hospitalization rates. Future studies should also include controlled designs and pre/post comparisons to evaluate whether the use of NephroGo leads to measurable improvements in CKD self-management and clinical outcomes.

Integrating electronic health records (EHR) could enhance data accuracy and enable automated monitoring. Additionally, qualitative research could explore user motivations and barriers in underrepresented groups, especially those with early-stage CKD or rural residents.

To ensure sustainability and maximize impact, health economic analyses are also warranted to quantify cost savings associated with improved self-management. Finally, co-designing app features with patients and clinicians across different healthcare systems may support broader implementation and cultural adaptation across Europe.

## 5. Conclusions

This study provides valuable insight into the long-term use of a digital health tool tailored for individuals with CKD. High levels of engagement, particularly among Stage 5 patients and middle-aged users, suggest that mHealth applications can effectively support dietary monitoring and self-management when designed with user needs in mind. While such tools benefit digitally literate and clinically engaged populations, existing disparities remain a concern, especially among older adults or those with lower health activation. These findings underscore the importance of usability, clinician endorsement, and patient-centered design in maximizing the reach and impact of digital interventions across the CKD continuum. mHealth tools hold promise for enhancing chronic disease management and reducing healthcare burdens. However, their integration should be supported by strategies that address varying levels of digital literacy, disease stage, and patient motivation.

## 6. Patents

The NephroGo application and all associated intellectual property are fully owned by Giedrė Žulpaitė and Karolis Vyčius.

## Figures and Tables

**Table 1:** Initial NephroGo mobile application user characteristics and usage patterns.

Category	Subcategory	Value
Total initial downloads		204
Active users		191 (93.6%)
Sex	Female	138 (67.6%)
	Male	66 (32.4%)
CKD	CKD > 10 years	111 (54.4%)
	Stage 5 CKD	52 (25.5%)
Renal replacement therapy	No therapy	81 (39.7%)
	Hemodialysis	59 (28.9%)
	Peritoneal dialysis	13 (6.4%)
Nutrition	Daily use of nutrition calculator	135 (66.2%)
	Avg. food items added/day	7 ± 1
	Exceed daily sodium norm	58 (30.2%)
	Exceed daily calorie norm	12 (6.3%)

**Table 2:** (1) Survey results: demographics, (2) Survey results: MARS score.

(1)		
	Survey Item	Value
	Number of respondents	32
Source of awareness about NephroGo	Healthcare professionals	14 (43.8%)
	Social media	11 (34.4%)
	Patients’ communities and dialysis centers	7 (21.8%)
Residence	Urban	26 (81.3%)
	Rural	6 (18.7%)
Educational Background	Higher education	18 (56.3%)
	Overall MARS score	4.09 ± 0.66
**(2)**		
	**Survey Item**	**Value**
MARS scores	Overall MARS score	4.09 ± 0.66
	Functionality score	4.27 ± 0.74
	Engagement score	3.81 ± 0.80
Highest rated function	Peritoneal dialysis	5.00 ± 0.00 (*p* = 0.0089)
Highest rated by	Stage 5 CKD patients	4.52 ± 0.52 (*p* = 0.0016)
Highest rated if	Recommended by a doctor	4.50 ± 0.68 (*p* = 0.039)

**Table 3:** NephroGo mobile application user characteristics after 4 years.

Category	Subcategory	Value
Total downloads		1670
Sex	Female	873 (52.3%)
	Male	797 (47.7%)
Age groups	0–10	19 (1.1%)
	11–20	26 (1.6%)
	21–30	91 (5.5%)
	31–40	277 (16.6%)
	41–50	199 (11.9%)
	51–60	239 (14.3%)
	61–70	201 (12%)
	71–80	102 (6.1%)
	81+	58 (3.5%)
	Unknown *	458 (27.4%)
CKD	Stage 1	142 (8.5%)
	Stage 2	130 (7.8%)
	Stage 3	257 (14.7%)
	Stage 4	245 (14.7%)
	Stage 5	455 (27.2%)
	Unknown stage *	441 (26.4%)
Duration of CKD, years	<1	465 (27.8%)
	1–5	457 (27.4%)
	6–10	229 (13.7%)
	>10	519 (31.1%)
Renal replacement therapy	No therapy	952 (57.0%)
	Hemodialysis	398 (23.8%)
	Peritoneal dialysis (automatic)	67 (4.0%)
	Peritoneal dialysis (manual)	149 (8.9%)
	Post-transplant	104 (6.2%)
Diabetes	No	1383 (82.8%)
	Type 1	123 (7.4%)
	Type 2	159 (9.5%)
	Unknown	5 (0.3%)

* ‘Unknown’ values reflect missing user-provided data during registration. Age defaults may result from users skipping birthdate entry, and CKD stage was self-reported and not mandatory. These missing values were excluded from subgroup comparisons but did not affect overall usage trend analyses.

**Table 4:** Food tracking.

Category	Subcategory	Value
Sex	Female	39 (59.1%)
	Male	27 (40.9%)
CKD stage	Stage 1	5 (7.6%)
	Stage 2	3 (4.5%)
	Stage 3	5 (7.6%)
	Stage 4	7 (10.6%)
	Stage 5	26 (39.4%)
	Unknown	20 (30.3%)

**Table 5:** Peritoneal dialysis tracking.

Category	Subcategory	Value
Sex	Female	9 (32.1%)
	Male	19 (67.9%)
Age categories	21–40	7 (25%)
	41–60	13 (46.5%)
	61–80	3 (10.7%)
	Unknown	5 (17.8%)

**Table 6:** Daily health tracking.

Category	Subcategory	Value
Sex	Female	119 (%)
	Male	97 (%)
CKD stage	Stage 1	9.1%
	Stages 2–4	36.3%
	Stage 5	45.5%
	Unknown	9.1%
Age categories	0–20	6 (2.8%)
	21–40	55 (25.5%)
	41–60	77 (35.6%)
	61–80	46 (21.3%)
	81+	5 (2.3%)
	Unknown	27 (12.5%)

## Data Availability

The data supporting the findings of this study are available from the corresponding author upon reasonable request. Due to privacy and ethical considerations, the dataset is not publicly available.

## References

[1] Hill N.R., Fatoba S.T., Oke J.L., Hirst J.A., O’Callaghan C.A., Lasserson D.S., Hobbs F.D.R. (2016). Global Prevalence of Chronic Kidney Disease—A Systematic Review and Meta-Analysis. PLoS ONE.

[2] Luyckx V.A., Tonelli M., Stanifer J.W. (2018). The Global Burden of Kidney Disease and the Sustainable Development Goals. Bull. World Health Organ..

[3] Kidney Disease Statistics for the United States—NIDDK. https://www.niddk.nih.gov/health-information/health-statistics/kidney-disease.

[4] Diamantidis C.J., Becker S. (2014). Health Information Technology (IT) to Improve the Care of Patients with Chronic Kidney Disease (CKD). BMC Nephrol..

[5] Self-Management Interventions for Adults with Chronic Kidney Disease: A Scoping Review|BMJ Open. https://bmjopen.bmj.com/content/8/3/e019814.

[6] Nunes J.A.W., Wallston K.A., Eden S.K., Shintani A.K., Ikizler T.A., Cavanaugh K.L. (2011). Associations among Perceived and Objective Disease Knowledge and Satisfaction with Physician Communication in Patients with Chronic Kidney Disease. Kidney Int..

[7] Siddique A.B., Krebs M., Alvarez S., Greenspan I., Patel A., Kinsolving J., Koizumi N. (2019). Mobile Apps for the Care Management of Chronic Kidney and End-Stage Renal Diseases: Systematic Search in App Stores and Evaluation. JMIR mHealth uHealth.

[8] Lunardi L.E., Le Leu R.K., Matricciani L.A., Xu Q., Britton A., Jesudason S., Bennett P.N. (2024). Patient Activation in Advanced Chronic Kidney Disease: A Cross-Sectional Study. J. Nephrol..

[9] Johnson M.L., Zimmerman L., Welch J.L., Hertzog M., Pozehl B., Plumb T. (2016). Patient Activation with Knowledge, Self-Management and Confidence in Chronic Kidney Disease. J. Ren. Care.

[10] R: The R Project for Statistical Computing. https://www.r-project.org/.

[11] Ebrahim Z., Glorieux G., Moosa M.R., Blaauw R. (2022). Effect of Simplified Dietary Advice on Nutritional Status and Uremic Toxins in Chronic Kidney Disease Participants. S. Afr. J. Clin. Nutr..

[12] Kosa S.D., Monize J., D’Souza M., Joshi A., Philip K., Reza S., Samra S., Serrago B., Thabane L., Gafni A. (2018). Nutritional Mobile Applications for CKD Patients: Systematic Review. Kidney Int. Rep..

[13] Bailey M.A., Dhaun N. (2024). Salt Sensitivity: Causes, Consequences, and Recent Advances. Hypertens. Dallas Tex 1979.

[14] Sin D., Harasemiw O., Curtis S., Iman Y., Buenafe J., DaCosta J., Mollard R.C., Tangri N., Protudjer J.L.P., Mackay D. (2022). Dietary Patterns and Perceptions in Older Adults with Chronic Kidney Disease in the Canadian Frailty Observation and Interventions Trial (CanFIT): A Mixed-Methods Study. Can. J. Kidney Health Dis..

[15] Park G., Choi S. (2023). The Effects of a Tailored Dietary Education Program for Older Adult Patients on Hemodialysis: A Preliminary Study. Healthcare.

[16] Hussein W.F., Bennett P.N., Abra G., Watson E., Schiller B. (2022). Integrating Patient Activation Into Dialysis Care. Am. J. Kidney Dis..

[17] Okoro R.N., Ummate I., Ohieku J.D., Yakubu S.I., Adibe M.O., Okonta M.J. (2020). Evaluation of Medication Adherence and Predictors of Sub-Optimal Adherence among Pre-Dialysis Patients with Chronic Kidney Disease. Med. Access Point Care.

[18] de Camargo Catapan S., Taylor M.L., Scuffham P., Smith A.C., Kelly J.T. (2025). Improving Consumer Trust in Digital Health: A Mixed Methods Study Involving People Living with Chronic Kidney Disease. Digit. Health.

[19] Kinshella M.-L.W., Boene H., Sevene E., Valá A., Sharma S., Vidler M., Magee L.A., von Dadelszen P., Munguambe K., Payne B.A. (2022). How Gender Influenced the Experience of Using a mHealth Intervention in Rural Mozambique: Secondary Qualitative Analysis of Community Health Worker Survey Data. Front. Glob. Womens Health.

[20] Hahn S.L., Hazzard V.M., Loth K.A., Larson N., Klein L., Neumark-Sztainer D. (2022). Using Apps to Self-Monitor Diet and Physical Activity Is Linked to Greater Use of Disordered Eating Behaviors among Emerging Adults. Prev. Med..

[21] Hussein W.F., Bennett P.N., Sun S.J., Reiterman M., Watson E., Farwell I.M., Schiller B. (2022). Patient Activation Among Prevalent Hemodialysis Patients: An Observational Cross-Sectional Study. J. Patient Exp..

[22] Li C., Wang H., Yuan J., Shi L., Chen Y., Gao Z., Zhao L., Oliveira A. (2025). Current Status of Older People with Chronic Diseases Adopting Digital Health Technologies: A Scoping Review. Digit. Health.

[23] Lee H.-Y., Ju M., Kang M., Lee H., Choi J., Oh J. (2024). Preparedness, Challenges, and Opportunities for Digital Intervention for Chronic Disease Management: A Qualitative Study in Rural Areas of South Korea. Health Syst. Reform.

[24] Kouri A., Gupta S., Straus S.E., Sale J.E.M. (2023). Exploring the Perspectives and Experiences of Older Adults with Asthma and Chronic Obstructive Pulmonary Disease Toward Mobile Health: Qualitative Study. J. Med. Internet Res..

